# A New Multivariate Optical Computing Microelement and Miniature Sensor for Spectroscopic Chemical Sensing in Harsh Environments: Design, Fabrication, and Testing

**DOI:** 10.3390/s19030701

**Published:** 2019-02-08

**Authors:** Christopher M. Jones, Bin Dai, Jimmy Price, Jian Li, Megan Pearl, Bill Soltmann, Michael L. Myrick

**Affiliations:** 1 Halliburton Energy Services, Houston, TX 77032, USA; ChrisJones.Jones@halliburton.com (C.M.J.); Bin.Dai2@halliburton.com (B.D.); jian.li@halliburton.com (J.L.); megan.pearl@halliburton.com (M.P.); Bill.soltmann@halliburton.com (B.S.); 2 Department of Chemistry and Biochemistry, University of South Carolina, Columbia, SC 29208, USA; myrick@mailbox.sc.edu

**Keywords:** multivariate optical computing, logging while drilling, permanent placement sensors, high temperature and pressure

## Abstract

Multivariate optical computing (MOC) is a compressed sensing technique with the ability to provide accurate spectroscopic compositional analysis in a variety of different applications to multiple industries. Indeed, recent developments have demonstrated the successful deployment of MOC sensors in downhole/well-logging environments to interrogate the composition of hydrocarbon and other chemical constituents in oil and gas reservoirs. However, new challenges have necessitated sensors that operate at high temperatures and pressures (up to 230 °C and 138 MPa) as well as even smaller areas that require the miniaturization of their physical footprint. To this end, this paper details the design, fabrication, and testing of a novel miniature-sized MOC sensor suited for harsh environments. A micrometer-sized optical element provides the active spectroscopic analysis. The resulting MOC sensor is no larger than two standard AAA batteries yet is capable of operating in high temperature and pressure conditions while providing accurate spectroscopic compositional analysis comparable to a laboratory Fourier transform infrared spectrometer.

## 1. Introduction

Real-time in-situ sensors now play an important role in not only the exploration and discovery of economic petroleum reservoirs but also in keeping oil and natural gas developments viable and maximizing their profitability over their production lifetime [1]. This fact resonates even more for those fields located offshore and in deep water. Initial fluid characterization during logging while drilling (LWD) operations allows asset evaluation and production design. Furthermore, asset owners effectively argue the value proposition of these sensors to enable remote monitoring and control of individual reservoir zones to help optimize production, diagnose issues, and update reservoir models and field development plans, that were initialized during logging evaluation, over the life of the reservoir [1,2]. Functionally the same types of sensors, measuring the same properties is needed for both LWD and permanent emplacement. Although temperature and pressure sensors are common for permanent emplacement and additional physical fluid measurements are common for LWD, advanced sensors capable of monitoring the physical and chemical properties of fluids are also needed [3,4]. These advanced intelligent options are necessary to address operational issues as well as to achieve the intended production mechanisms over the lifetime of the reservoir. As a result, a higher capital investment is required upfront, however this far outweighs the alternative risks and expenditures associated with wellbore shut-ins and mechanical interventions during production. This cost/value tradeoff is increasingly more important for deep offshore wells that necessitate higher pressure and temperature reliability requirements for sensors. In such conditions, permanently placed sensors are expected to last for 20 years, and their associated electrical components need to be deemed reliable and capable of withstanding temperatures up to and exceeding 230 °C while their valves and seals need to demonstrate sufficient yield strength necessary to withstand pressures as high as 138 MPa. Furthermore, these conditions require a reduced sensor footprint for LWD operations or to fit within the completion tubulars of a well. LWD environments also experience significant vibration and shock as the bottom hole assembly penetrates and navigates rock formations. Due to the fact that the active spectrometer section of the current sensor is a solid-state element and the sensor has no moving parts, the device is highly suited for LWD environments.

This paper highlights the design and component lifetime testing of a sensor intended to be utilized in LWD operations or be permanently placed downhole in petroleum wells. The sensor is designed to monitor carbon dioxide (CO_2_) concentration levels in an oil or a water host matrix. The concentration of carbon dioxide, which can now be revealed by LWD measurements, is in some cases the critical economic factor that decides whether to complete a reservoir section for production. Enhanced oil-recovery methods are often employed to extract (on average) 30 to 60% of the reservoir’s original oil once the primary methods attributable to natural pressure and artificial lift have been exhausted [5,6]. Of the different enhanced oil-recovery methods, CO_2_ gas injection is one of the more widely used approaches [2]. This method relies on a separate injection well to pump CO_2_ into the reservoir, thereby reducing the viscosity of the oil present in the reservoir and creating a “pressure front” to push the oil toward a production well. Ideally, the injection rate creates a planar wave front that pushes the oil uniformly toward the production well. If the injection rate is not optimized to the specific reservoir pressure and temperature conditions, the CO_2_ can leach through (ahead of the oil) toward the production well [7]. Therefore, a permanently placed downhole CO_2_ sensor is needed to optimize the injection/production rates and identify if/when CO_2_ permeates beyond the planar pressure wave-front. In such a case, the operator can choke back on the injection/production rates. Additionally, an array of sensors placed at different stations can identify a specific location from where the issue is originating, and that source can be shut in. It is worth noting that other photometric-based CO_2_ sensing devices exist. Such devices may have a larger selectivity by exploiting attenuated total reflection techniques focused on the fundamental CO_2_ vibration band in the mid-infrared (mid-IR) near 4275 nm using a diode or LED laser. However, these devices are not viable as permanently placed sensors in harsh downhole environments.

One suitable candidate technique for downhole fluid identification in harsh oil and gas environments is multivariate optical computing (MOC). MOC is a compressed sensing technique used to extract compositional information from a straightforward broadband optical transmission measurement [8,9]. A typical oil and gas optical spectrum consists of a multitude of interfering absorption peaks characteristic of all the chemical species present. The magnitude of these absorption peaks is governed by the linear compositional relationship of Beer’s law [10]. This equation, expressed in vector notation, can be used to discover information about the concentration (c) of a single optically active component within the sample:

(1) $${\left(L\cdot \varepsilon \right)}^{-1}\cdot A=c$$

where *A(λ)* is the wavelength-dependent absorbance of light, *ε(λ)* is the molar absorptivity coefficient, and L is the optical path length. (*Lε*)^−1^ can be defined as a regression vector, *R_v_*. Classical least squares, principal component (PC) regression, partial least squares (PLS), and alternating least squares are a few of many different chemometric modeling methods used to find an appropriate regression vector [10,11]. Most importantly, a true multivariate analysis identifies a regression vector that is unique to a specific analyte or characteristic of interest while remaining orthogonal to all interferents. Once a regression vector is determined, the dot product can be performed between the regression vector and the spectrum, I, of a sample to determine the concentration:

(2) $$C=R_{v}\cdot I$$

MOC sensors eliminate the extreme difficulty of placing a laboratory spectrometer downhole and also circumvent the complex offline chemometric analysis by directly encoding the regression vector into an optical transmission filter, hereafter referred to as a multivariate optical element (MOE) [12]. In doing so, MOEs offer an optical platform that performs both the sensing and optical computation in a single step to extract chemical information. The spectral response for the regression vector is typically designed using optical interference principles to determine a plurality of optical thin-film layers consisting of various materials, whose index of refraction and thickness can vary between each layer. The layers can be strategically deposited and sized so as to selectively pass and reflect predetermined fractions of light at different wavelengths, which are configured to substantially mimic the regression vector. The classical regression of the dot product with the regression vector and the sample spectrum is then performed by placing the filter directly in the optical path. As a result, the convoluted output light intensity of the light source, sample, and MOE conveyed to the detector can be directly related to an optically active physical or chemical property of the substance. No spectrum needs to be collected, and the analog computation occurs at the speed of light. Since a spectrum is never collected, MOC is not a spectroscopic technique, but rather falls into the category of “photometric analysis”.

Various literature reports have detailed the successful use of MOC for in-situ downhole chemical analysis [12,13,14,15,16,17,18,19]. However, the existing (legacy) sensor configuration is not suited for permanent placement in harsher conditions, as previously outlined. To this end, a new sensor was developed with a significantly decreased footprint, no moving parts, and the ability to withstand high temperatures and pressures. This paper details the design and development of this sensor and the testing necessary to ensure its success. More specifically, specific modules and results are discussed, showing how the legacy sensor was transformed in size and how the testing was performed to ensure it maintained accuracy and sensitivity while operating at high temperature and pressure over an accelerated lifetime projection of 20 years.

## 2. Materials and Methods

**Figure 1:**
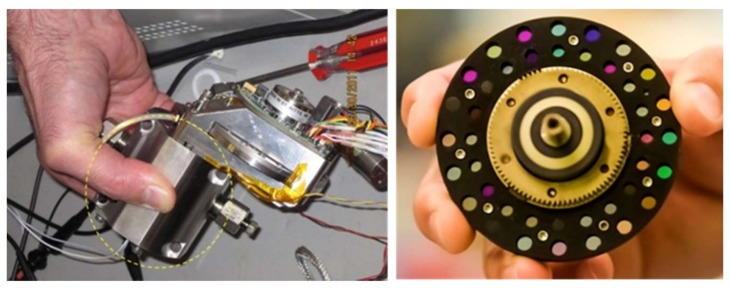
Multivariate optical computing (MOC) sensor body (**left**) and close up of multichannel wheel (**right**).

The goal of this work was to modify an existing MOC sensor while ensuring a significantly reduced footprint and maintaining the capability of operating in harsh environments. Additionally, this new sensor needs to be qualified for 20+ years of in-situ operation. Figure 1 shows the legacy sensor. The sensor body (Figure 1, left) has an area of ~9 × 14 cm^2^ and consists of a broadband lamp that directs the light through a fluid flow cell, an MOE, and onto an optical (thermopile) detector whose output relays real-time concentration information. The sensor body also houses an MOE carousel (Figure 1, right), which rotates (powered by a rotational motor) various MOEs into the optical beam path to measure a different analytes or properties associated with that specific MOE. By rotating the carousel, multiple fluid properties can be interrogated almost simultaneously. More importantly, this implementation allows for adding or changing different MOEs based on the desired measurement or fluid environment. The MOE carousel, which is ~7.5 cm in diameter, is configured with an inner and outer row capable of holding 20 MOE/reference pairs each.

In an effort to satisfy the new sensor design requirements and miniaturize the sensor body geometry for a permanently placed downhole sensor, the MOE carousel (along with the motor and associated electronics) was substituted with a sensor design that delivers only a single real-time answer product. In practice, an MOC sensor requires a minimum of only two separate detector channels for an accurate measurement. Along with the MOE designed for a specific analyte of interest, a separate channel is also required to normalize the signal-to-unit variance and help eliminate perturbations caused by light intensity fluctuations and drift. A neutral density filter band passed over the same spectral region is typically used for this purpose. Furthermore, fairly recent developments in design and fabrication principles of MOEs demonstrate superior measurement accuracy and sensitivity when two transmission patterns (i.e., the MOE and the reference) are simultaneously optimized to work together as a single regression vector. In such a configuration, the reference bares some of the complexity of the regression vector and, hence, is no longer only a reference but part of the regression vector. As such, the new configuration is called a dual-MOE design. The design flexibility gained by using more than one MOE enables finding an answer product solution that might prove difficult to solve using a single-MOE approach. The design complexity of each of the two MOEs can be significantly relaxed and more easily manufactured with a lower probability of error and variability. Finally, superior accuracy in the predictive performance is realized as a regression vector can be achieved that has a much higher frequency structure compared to a single-MOE transmission profile and can therefore target the shape of the ideal theoretical regression vector. Since all MOE configurations inherently require a two-channel measurement, the new configuration experiences no complexity issues with respect to the previous configurations [18,20,21]. With these considerations in mind, a minimum of two separate MOEs and one neutral density channel are required for the application of a miniaturized sensor. The thermopile detector was therefore replaced with a four-channel quad detector. This offers a fixed beam path wherein all channels are measured simultaneously after the light passes through the fluid sample. Two of the detector channels are dedicated to the MOEs, one channel is committed to a neutral density filter for normalization, and an open or dark channel is used for auto-zero corrections.

The solid-state quad detector has four active regions, each with an area of 1 mm^2^. Each active region is separated from its neighbor by a “street distance” of 0.1 mm. One obvious concern with the miniature size of the quad element array is the cross-talk between neighboring channels. This design application requires that each channel operate independently of, yet simultaneously with, the other channels. Therefore, it is necessary that light passing through the fluid sample and exiting from an MOE (or neutral density filter) not contaminate a neighboring channel. Optical ray-tracing simulations were performed with a commercial software package to assist the design process and optimize the new sensor body around the full optical path to help maximize accuracy and sensitivity. One exercise of the ray-tracing simulations indicated that each MOE needs to be installed and aligned accurately to cover the exact area of the corresponding quad array, and the alignment error in the installation cannot exceed that of the street distance (i.e., 0.1 mm). Compounding errors are introduced if the MOEs are placed farther away from the surface of the detector active area. For situations where the MOE is placed at a distance greater than the street distance away from the active area, the results of the simulations showed that a light incident at non-normal angles on the MOEs will also exit the MOE at a greater angle and can interact with a neighboring detector channel. Cross-contamination between channels would negatively impact the performance of the design and should therefore be avoided by placing the MOE as close to the active region as possible. The micro-scale nature of these detector array channels also poses issues with respect to how the MOEs are prepared for installation after fabrication. The MOEs are traditionally fabricated on 2.54-cm-diameter glass substrates before coring them down to the 6-mm diameter necessary to rest in the housing unit of the carousel. For this miniaturized sensor and alignment with the quad detector, the fabricated MOEs would therefore have to be cored down to 1 mm^2^, which would introduce additional complications with respect to edge effects, handling, alignment, and securing the MOE in place.

**Figure 2:**
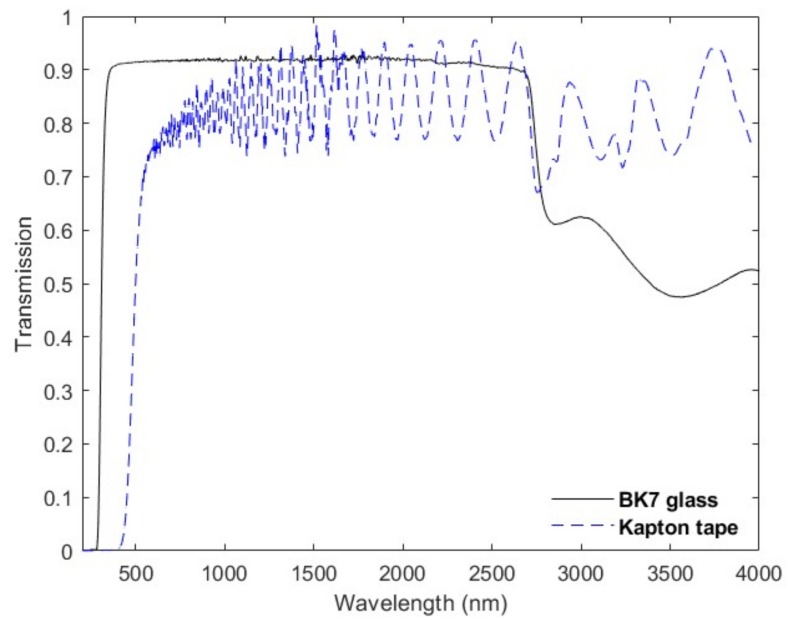
Transmission spectra of BK7 glass substrate (solid black) and Kapton^®^ tape (dashed blue) over visible to infrared (IR) wavelengths (400 to 3500 nm).

To avoid this list of concerns regarding the accurate alignment of an MOE to its corresponding active detector region, a novel approach of directly adhering the MOE to the detector surface was developed. The traditional glass substrate used for MOE fabrication was replaced with Kapton^®^ tape. Several characteristics make Kapton tape useful as the host substrate. First, it is durable and can withstand harsh conditions downhole as well as the high temperatures and plasma environment within the MOE deposition chamber. Second, Kapton tape is a flexible adhering polyimide-based material, allowing it to be directly applied to the active region of the detector. Third, Kapton tape is transparent over the wavelength region of interest for these MOEs. Figure 2 plots the transmission spectra for both the traditional BK7 glass substrates used for MOC legacy sensors and Kapton tape. Although Kapton tape does not extend as deep into the visible spectrum (cut-on ~500 nm), it does remain optically flat up to ~2500 nm and offers sufficient light throughput to design and fabricate an MOE.

To test this concept, laminated thin-films of Si and SiO_2_ were sequentially deposited on Kapton tape in a thin-film vacuum deposition chamber. Since MOEs possess the highly stringent sensitivity and accuracy design requirements necessary to achieve the desired regression vector pattern, several advanced thin-film deposition techniques have been developed and customized to realize the intended transmission spectrum. The MOEs were fabricated using a custom-built ion-assisted electron-beam vacuum deposition process. For process control, the system uses an in-situ J.A. Woollam Co. ultraviolet (UV), visible (VIS), and near infrared (NIR) spectroscopic ellipsometer, a near-infrared (NIR) and mid-IR Fourier-transform infrared (FTIR) spectrometer, an optical monitor system, a quartz crystal monitor, and custom software designed specifically for the fabrication of the MOEs. The quartz crystal monitor and the optical monitor system are used to control the deposition rate and end-of-layer termination triggers. The spectroscopic ellipsometer and FTIR spectrometers are used to measure the optical constants, thickness, and transmission spectrum of the deposited material during fabrication. Real-time feedback control of layer thicknesses and optical constants allows for adjustment of the remaining deposition process to accurately match the target design transmission spectrum and help achieve the intended MOE design requirements. An ion source was also determined to be crucial during the deposition process to create high-density, repeatable-material optical constant, moisture-stable, and low-porosity films. These thin-film properties are important for the MOE to work successfully in a downhole environment, where high temperature and humidity can cause hysteresis in the material optical constants and therefore in the transmission spectrum.

## 3. Results

The calibration spectral dataset used for the CO_2_ design process was gathered from an in-house pressure–volume–temperature (PVT) fluid spectra database. This database encompasses numerous fluid spectra from various oil and gas mixtures over a range of reservoir formation temperatures and pressures, along with the corresponding compositional density and molecular weight information. For this design, 291 fluid samples were chosen, representing light and medium (as defined by the American Petroleum Institute, API) API gravity oils. Attention was focused on the concentration ranges for not only the analyte of interest but also the interferents to help ensure that they were representative of the downhole reservoir conditions. For the given dataset, the CO_2_ density range was 0.106 g/cc.

**Figure 3:**
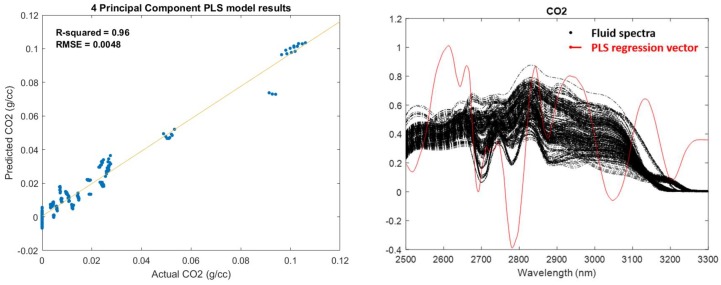
Partial least squares (PLS) model prediction plot identifying a theoretical PLS calibration error of 0.0048 g/cc (**left**) and corresponding four-principal component PLS regression vector (**right**, red solid line) of representative CO_2_ fluid spectra (right, black dotted line).

Figure 3 shows the results of the PLS analysis for all CO_2_ samples and their respective spectra.

The PLS analysis identifies a root-mean-square error of cross-validation (RMSECV) of 0.0048 g/cc (or 4.5% of the range) using a four-principal component PLS model. This helps establish a lower limit of what could be expected from the MOE design process and will help steer the optimization routine toward a global minimum. While evaluating certain designs, solutions significantly greater (e.g., 2×) than the PLS error limit can be discarded. In the event that the dataset was not adequately defined and/or had sufficient rank, the PLS analysis would yield a poor RMSECV that would not meet acceptable design criteria and indicate that the calibration dataset needs to be revisited. Along with the theoretical performance limits, the PLS analysis also provides the four-principal component PLS-based regression vector. The regression vector can also be used to help direct the optimization routine and identifies the spectral regions most important for the analyte of interest. From the right-hand plot of Figure 3, important spectral regions between ~2650 to 2800 nm can be identified that are expected to significantly contribute to the CO_2_ measurements.

The design optimization process for the CO_2_ MOE relies on the chemometric analysis method of multiple linear regression (MLR). The purpose of regression analysis is to construct a model that predicts the statistical relationship between a scalar-dependent response variable, “*Y*” (i.e., the concentration), and an independent variable, “*X*” (i.e., the multiwavelength spectra). Provided the dependent variable, *Y*, is continuous and has a normal distribution, a linear regression analysis model can be employed to characterize their statistical relationship. In the context of multivariate optical computing, this statistical relationship can be captured in the form of the optical regression vector used in Equation (2):

Regression Vector, R_V_ = α × T_a_ + β × T_b_ (3)

where *α* and *β* are the regression coefficients obtained by MLR using the method of ordinary least squares and *T_a_* and *T_b_* are the two separate MOE transmission spectrum profiles. The MOE design method therefore searches for the optimal MOEs (*T_a,b_*) that, when multiplied by the regression coefficients (*α* and *β*, respectively), produce an optical regression vector. Ideally, this regression vector should be similar to the PLS regression vector, such that, when combined as the inner product with the fluid spectra, it produces a predicted concentration with minimal residual error with respect to the true concentration.

To help ensure that a global minimum in the prediction error is identified, multiple (on the order of hundreds) initial MOE seed designs are generated during the design process having a random number of layers and random thicknesses. Next, each MOE seed design is projected against the calibration spectral dataset by obtaining the dot product between the MOE transmission profile and the spectral data. The dot product between each MOE transmission profile and the convolved spectral dataset represents the virtual detector response. An MLR model can then be established which consists of the two virtual detector responses and the measured CO_2_ concentration. The result of this MLR model yields two regression coefficients (*α* and *β*) that can be applied to each MOE transmission profile to define the optical regression vector. The optimization routine then continues this process by iteratively changing the layer thicknesses of each MOE to achieve new MOE spectra. The optimization routine is terminated when the resulting optimized optical regression vector produces a minimum in the objective function. The objective function (MSQ) for the optimization routine was defined as:

(4) $$MSQ=\sqrt{\frac{1}{n}\sum_{i=1}^{i=n}\sigma_{i}^{2}},$$

(5) $$\mathrm{Where}\;\sigma_{i}=\left|y_{i}-\hat{y_{i}}\right|,\;\mathrm{iff}\;\left|y_{i}-\hat{y_{i}}\right|>\frac{1}{SNR}\sqrt{\alpha^{2}*Det_{A}^{2}+\beta^{2}*Det_{B}^{2}},$$

(6) $$\mathrm{Else},\;\sigma_{i}=\frac{1}{SNR}\sqrt{\alpha^{2}\times Det_{A}^{2}+\beta^{2}\times Det_{B}^{2}}.$$

where *n* is the number of calibration spectral samples, ‘$\hat{y}$’ is the estimated value representing the concentration of the property of interest (‘*y*’), SNR is an empirically determined signal-to-noise ratio used to constrain the design solutions to meet acceptable real-world sensor sensitivity criteria, and *Det_A,B_* is the virtual detector response for MOEs A and B, respectively. This specific design objective function was derived to weight the sensor response for individual calibration spectral data samples resulting from their error associated with either accuracy or sensitivity. For example, if the accuracy ($\left|y_{i}-\hat{y_{i}}\right|$, Equation (5)) for a given sample is considerably poor, then the design evolution will evolve to reduce this error. On the other hand, if the sensitivity (or noise, Equation 6) for a given sample outweighs the error attributed to accuracy, then the design evolution will emphasize this contribution. Essentially, the objective function (MSQ) is derived to optimize a pair of MOEs such that both the error associated with accuracy and that associated with sensitivity are minimized. MSQ scales linearly with accuracy, and typically the minima for MSQ will also be the most accurate design. However, another design criterion is to consider the regression coefficients *α* and *β*. These are correlated to the sensitivity of the sensor, as large values for either of these coefficients will serve to amplify noise and place less emphasis on the MOE itself. Ideally, one would want these coefficients to be as small as possible, such that the emphasis is on the MOE and not the weighted coefficients. This is accounted for in Equation (6), which serves to minimize this function with respect to both of these coefficients. There is no actual advantage of optimizing a design solely focused on either accuracy or sensitivity, since a sensor response is not valuable if the accuracy is close to the PLS limit for a given calibration sample but is completely insensitive for other samples. Finally, it is worth mentioning that other possible options exist for the design optimization objective function, including the net analyte signal (NAS), sensitivity, or matching the PLS regression vector [22].

**Figure 4:**
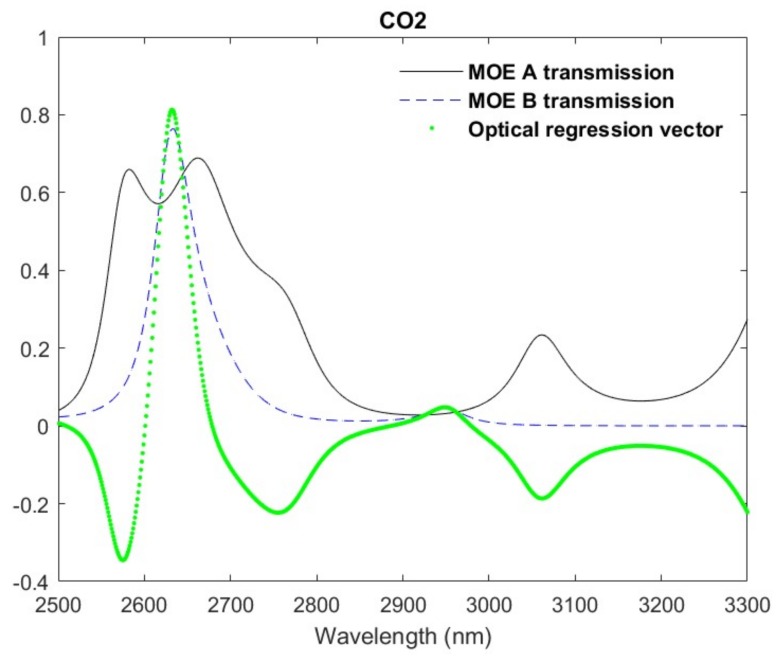
Dual-core transmission profiles for CO_2_.

Figure 4 shows the results of the design optimization process for the CO_2_ MOE design. Both transmission profiles and the corresponding optical regression vector are plotted for the best performing (i.e., lowest MSQ) design. The width and frequency of the individual MOE transmission peaks and valleys are dictated by the thin-film structure and have a limit to the fidelity of their features. These features will change, obviously, for different thin-film structures of the MOE, however it is typical for the resolution to be on the order of 50 nm—an order of magnitude larger than the spectral fidelity observed for the PLS regression vector in this spectral region. Figure 4 also plots the optical regression vector, which is obtained by linearly combining each transmission profile with its corresponding regression coefficient determined during the design optimization process. As a result of the linear combination, the optical regression vector is able to mimic the higher spectral fidelity of the theoretical PLS regression vector.

**Figure 5:**
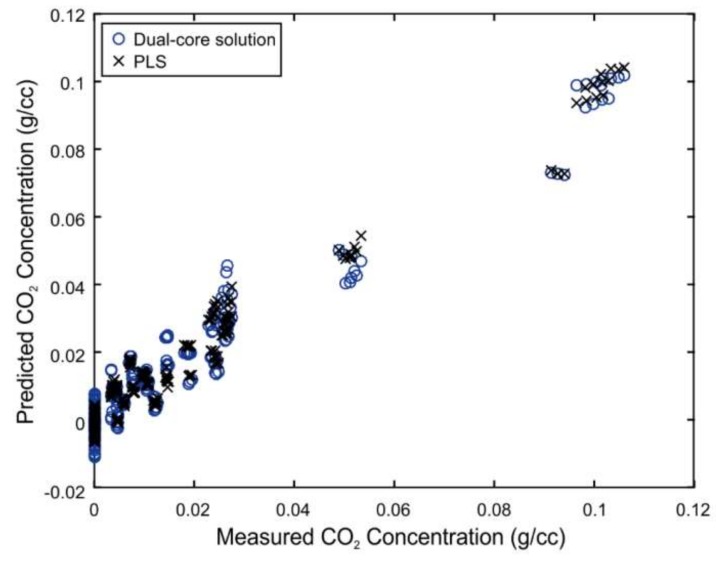
Comparison of predicted results between the dual-core optimized design and PLS.

Figure 5 demonstrates the close agreement between the predicted results (RMSECV = 0.0057 g/cc, or 5.4% of the range) achieved with the optimized CO_2_ MOE sensor and that of the PLS analysis.

**Figure 6:**
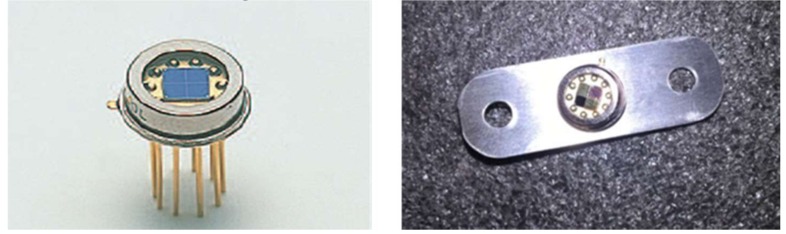
Commercial quad-array detector (**left**) and after three active area arrays are covered with different multivariate optical elements (MOEs) fabricated on Kapton tape (**right**).

As a test of fabrication capability, assembly, and component testing, three different MOEs were fabricated on Kapton tape substrates and then transferred directly to the active area surface of three different quad detectors, as shown in Figure 6 (right). The fourth channel was left open as a reference. Both options were investigated where the MOE/Kapton tape was scribed before adhering to the detector, as well as directly adhering the tape to the active region and then scribing away the excess. For component testing, the visible spectral region was chosen because of the simplicity of photometric measurements relative to the IR region.

**Figure 7:**
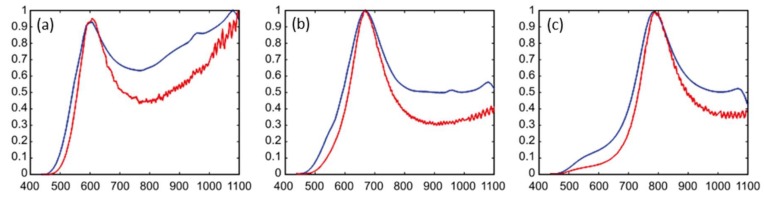
Normalized ratio of detector response (red) for each quad-detector array element (**a**–**c**) compared with the corresponding transmission spectrum (blue) of the MOE deposited directly on the Kapton tape substrate.

Figure 7 plots the normalized (with respect to the open channel) ratio of each quad-detector response (after installing the adaptable MOE) and is compared with the corresponding MOE/Kapton tape transmission spectrum obtained after deposition and before mating with the quad detector. The main peak features of the MOE transmission spectra were successfully convolved with the detector responses. Deviations in the spectral regions away from the main peak wavelengths can be corrected by a routine calibration process. Applying the MOE/Kapton tape directly to the active area surface did not impede the function of the detector nor distort the overall spectroscopic response of each channel.

**Figure 8:**
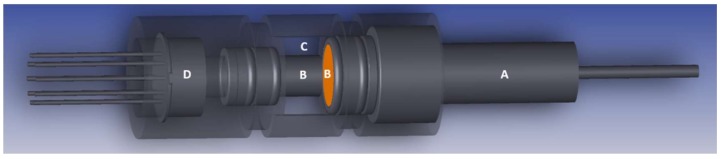
Cross-sectional schematic of microsensor. (**A**) light source, (**B**) Sapphire window (**right**) and rod (left), (**C**) fluid flow path, and (**D**) quad photodetector.

Elimination of the MOE carousel and adhering the MOEs directly to the quad-detector active arrays established the detector diameter as the main sensor body component dictating the upper limit of the sensor size. Further reductions in the sensor body geometry were achieved by replacing the lamp with a smaller 0.64-cm-diameter smooth-sided housing with the optical axis aligned to the mechanical axis of its base. Optional axial adjustments are possible with this lamp to optimize the light distribution and pattern. The electronics are housed external to the cell body but packaged in a way such that the entire microsensor and electronics body is no bigger than two AAA batteries. Figure 8 illustrates a cross-sectional view of the newly adapted microsensor. Light originates from the right-hand side (A), where the lamp is embedded in the cell housing. The 1-mm fluid flow compartment (C) is sandwiched between the two sapphire windows (B) and is designed to provide sufficient flow through the cell without allowing turbulence or flow interruption. The light interrogates the sample through the sapphire windows before passing to the quad detector (D) with the MOEs adhered to each surface.

## 4. Discussion

Reducing the overall sensor body size necessitated the use of a new light source, photodetector, MOE fabrication process, and an optical beam path that interrogates the fluid sample. It is therefore an obvious next step to investigate how these changes impact the performance of the new miniaturized sensor. This section benchmarks the new sensor sensitivity vs. the legacy sensor as well as prototyping the reliability of the new design against the additional performance requirements of operating at high temperature and for an extended lifetime. 

Noise-equivalent-power (NEP) is a commonly accepted metric to subjectively quantify a photodetector’s sensitivity or the power generated by a noise source. NEP can be defined as the input signal power that results in an SNR of 1 in a 1-Hz output bandwidth. Therefore, the photosensitivity of the new miniaturized sensor can be quantitatively compared with the legacy sensor by convolving each of their respective component contributions and scaling this by the factory reported value for NEP of both the silicon (Si) photodiode [NEP ~9 × 10^−15^ W/(Hz)^1/2^] and thermopile detectors [NEP ~1 × 10^−9^ W/(Hz)^1/2^], respectively. Specifically, radiometric measurements were performed on both the new lamp and detector to obtain their respective intensity profiles. These spectroscopic profiles were then convolved with the transmission spectra of the bandpassed MOEs and sapphire windows to obtain a relative transmitted light intensity. Finally, this efficiency factor was scaled by the operating power of the lamp and used as an input to the ray-tracing software to simulate the efficiency of how many photons were reaching the detector. The NEP of each detector could then be scaled by the simulated light throughput for each sensor. For the ray-tracing simulations, the exact sensor body geometry was imported into the software package directly from the SolidWorks^®^ software (registered trademark of Dassault Systèmes, Vélizy-Villacoublay, France). For a conservative SNR of 200, the results of the simulations determined that the solid-state Si detector micro-sized sensor yielded approximately seven orders of magnitude greater sensitivity than the legacy sensor with the thermopile detector. These simulation results are to be expected considering the given higher resolution, sensitivity, and NEP of solid-state detectors. These results additionally confirm that the changes in the sensor body beam path do not negatively impact performance when reducing the sensor geometry.

With the new process modification applied to depositing the MOEs directly onto Kapton tape and adhering to the detector active area surface, a concern is introduced that their optical properties might degrade within a short time period or at significantly elevated temperatures. Furthermore, for the application of the permanently placed downhole miniaturized sensor, the sensor is required to work for a minimum of 20 years. An accelerated lifetime test was performed to address these reliability concerns. The test protocol was based on a well-accepted reliability testing protocol used by the semiconductor industry for the defects generated in Si-based devices (Si is also a key component used in the fabrication of the MOEs). Based on the Arrhenius equation, an acceleration factor can be introduced as:

(7) $$\alpha =e^{\frac{E_{a}}{k}\left(\frac{1}{T_{a}}-\frac{1}{T_{s}}\right)}$$

where *E_a_* is the activation energy, *k* is Boltzmann’s constant, *T_a_* is the expected operating temperature during use, and *T_s_* is the stress temperature. In this work, the sensor operation temperature is approximately 120 °C, the stress temperature was selected to be 200 °C, and an assumed activation energy of ~0.7 eV was used based on other Si-based device studies. The acceleration factor can then be calculated to be ~33, meaning that a one-month test would suggest performance over 33 months. Successful accelerated lifetime testing was conducted for the MOE/Kapton process, a visible light source, an IR light source, a Si (visible) photodiode detector, an indium gallium arsenide (InGaAs) (IR) detector, and a broadband thermopile detector [23,24].

**Figure 9:**
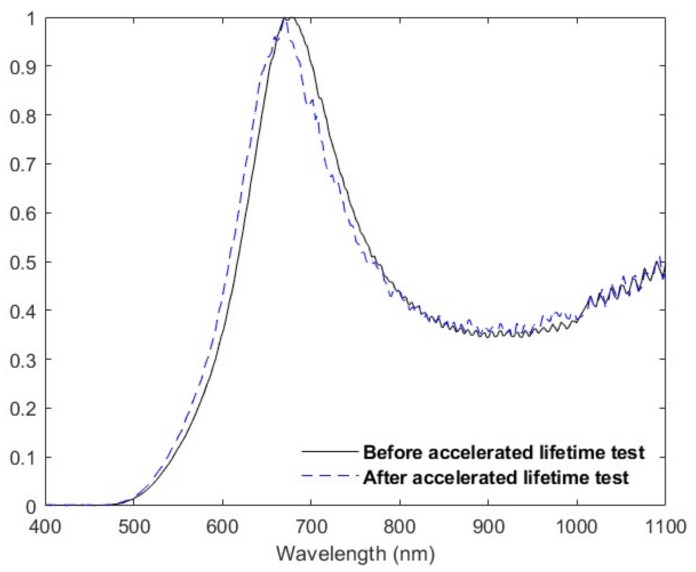
Comparison of MOE transmission spectrum before and after accelerated lifetime testing.

Figure 9 provides the results of the accelerated lifetime test comparing the normalized optical transmission of a high-adaptability MOE before and after 1845 h of testing. Since the test temperature was higher than the fabrication temperature, there was a negligible annealing effect that occurred at the beginning of the test. However, continued accelerated lifetime testing at a constant 200 °C over an extended eight-month period identified no degradation in the spectrum fidelity or hysteresis with initial testing. An eight-month accelerated lifetime test would translate to an equivalent downhole time of ~22 years.

## 5. Conclusions

A miniaturized multivariate optical computing sensor was developed as a solution for downhole fluid characterization in high-pressure and -temperature environments. This device now enables LWD operations and permanent-emplacement remote monitoring. With a temperature qualification of up to 230 °C and pressure qualification of up to 138 MPa, this sensor is suitable for numerous downhole environments. The sensor body geometry was significantly modified from a much larger legacy sensor used in downhole logging operations. To accomplish this, the versatility of offering multiple analyte measurements from a single sensor had to be replaced with a sensor offering only a single real-time analyte measurement. A new approach for fabricating optical filter elements directly on flexible adhering Kapton tape substrates and subsequently sticking each to the active area of a detector array was developed. Making these sensor component changes allowed the sensor body geometry to be reduced to the size of two AAA batteries. Sensitivity studies using optical ray-tracing software demonstrated significantly improved performance compared to the larger sensor. Accelerated lifetime testing indicated that the individual components can withstand the high-temperature and -pressure environments required of a permanently placed downhole sensor for at least 20 years.
